# Au_20_(^*t*^Bu_3_P)_8_: A Highly Symmetric Metalloid Gold Cluster in Oxidation State 0

**DOI:** 10.1002/anie.202206019

**Published:** 2022-07-28

**Authors:** Florian Fetzer, Nia Pollard, Nadine C. Michenfelder, Markus Strienz, Andreas N. Unterreiner, Andre Z. Clayborne, Andreas Schnepf

**Affiliations:** ^1^ Institut für Anorganische Chemie Universität Tübingen Auf der Morgenstelle 18 72076 Tübingen Germany; ^2^ Department of Chemistry and Biochemistry George Mason University 4400 University Drive MSN 3E2 Fairfax VA 22030 USA; ^3^ Institut für Physikalische Chemie Karlsruher Institut für Technologie Fritz-Haber-Weg 2, Geb. 30.44 76131 Karlsruhe Germany

**Keywords:** Gold, Jellium Model, Metalloid Cluster, Oxidation State, Structure

## Abstract

Metalloid gold clusters have unique properties with respect to size and structure and are key intermediates in studying transitions between molecular compounds and the bulk phase of the respective metal. In the following, the synthesis of the all‐phosphine protected metalloid cluster Au_20_(^*t*^Bu_3_P)_8_, solely built from gold atoms in the oxidation state of 0 is reported. Single‐crystal X‐ray analysis revealed a highly symmetric hollow cube‐octahedral arrangement of the gold atoms, resembling gold bulk structure. Quantum‐chemical calculations illustrated the cluster can be described as a 20‐electron superatom. Optical properties of the compound have shown molecular‐like behavior.

## Graphical abstract

A theoretically long‐studied Au_20_ cluster was synthesized by a wet chemistry approach, solely stabilized by phosphine ligands. The presented superatom cluster possesses a high symmetrical structure and an oxidation state of the gold atoms of ±0.

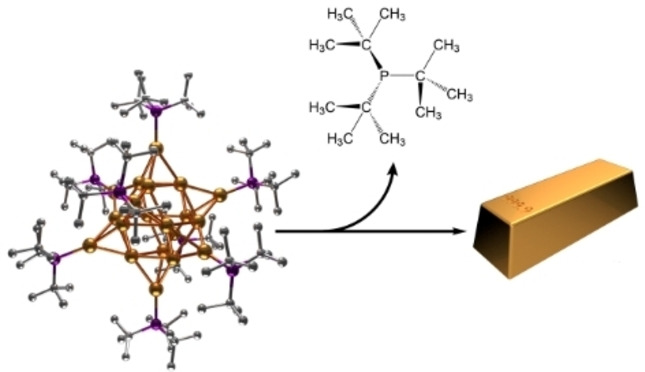

Since the first synthesis of gold nanoparticles within the purple of Cassius^[1]^ and the investigations of the different colors of gold nanoparticles by Faraday,^[2]^ gold nanoparticles and metalloid gold clusters are synthesized via the reduction of an Au^I^ or Au^III^ precursor like (PPh_3_)AuCl or HAuCl_4_ respectively.^[3]^ Other investigations in gold chemistry are either based on elemental gold or on compounds that have gold atoms exhibiting a zero oxidation state. The formation of larger gold compounds, consisting of up to hundreds of gold atoms, is achieved by the reduction of gold precursors.^[4]^ With increasing size of these compounds, the average oxidation state of the gold atoms gradually decreases, but does not reach zero.

Nanoclusters and nanoparticles represent an intermediate state between molecular gold compounds and the bulk phase. Within this size regime, these systems exhibit a manifold of different structures that resemble the elemental gold fcc‐lattice as well as novel polyhedral structures.^[5]^ Metalloid clusters of the general formula M_*n*_R_*m*_ (*n*>*m*, M=metals like Au, Ag, Al etc., R=organic substituent like N(SiMe_3_)_2_ or ligands like PPh_3_), which are actually often named “metal clusters” or “metal nanoclusters” or “monolayer protected metal clusters”, are ideal model compounds to illuminate the transition area from molecular compounds to bulk materials.^[6]^ Interestingly, many fascinating unique properties arise due to the small size and are used in applications. For example, catalytic activity,^[7]^ biomedical applications^[8]^ and unique electronic properties^[9]^ continue to drive research on metalloid gold clusters. Also, these studies illustrated that the structure and composition of metalloid gold clusters play a role in the precise observed property including state of oxidation.^[10]^

The precise structures of metalloid clusters can be determined by single‐crystal X‐ray structure analysis, which can also aid to study the transition from molecular to metallic compounds.[^11^, ^12^] The requirements to define clusters as metallic or molecular compounds are often based on optical properties, examined by transient and UV/Vis absorption spectroscopy, as well as measurements of plasmonic activities.^[13]^ Further, the arrangements of the gold atoms within the cluster cores are of importance as larger clusters tend to exhibit bulk like structures, consisting of fragments or deviations of the face‐centered cubic (fcc) arrangement present within elemental gold.[^14^, ^15^]

Since the first crystallographic characterized metalloid gold cluster Au_11_(PPh_3_)_7(_SCN)_3_ ^[16]^ a manifold of structures have been discovered. Among them are some landmark findings which furthermore increased interest in gold cluster chemistry such as the first multi‐shell gold cluster Au_102_(*p*‐MBA)_44_ ^[17]^ or the to date largest known metalloid gold cluster Au_279_(SPh‐^*t*^Bu)_84_.^[14]^ All yet reported gold clusters, as well as molecular gold compounds, have an average oxidation state of the gold atoms of ≠0 in common.

Gold clusters built from gold atoms in the oxidation state of ±0 are almost exclusively found in the gas phase, generated for example by laser vaporization.^[18]^ One intriguing example is an Au_20_ cluster that was extensively studied, by theoretical calculations,^[19]^ direct imaging by electron microscopy,^[20]^ as well as by gas phase studies including spectroscopic experiments.[^18^, ^21^] Although effort was put towards wet chemical approaches to isolate an Au_20_ cluster built solely from gold atoms in the oxidation state ±0 none was successful to date.^[22]^ Nevertheless, these studies yielded the all phosphine protected Au_22_ cluster Au_22_L_6_ (L=1,8‐bis‐(diphenylphosphino)‐octane),^[23]^ which represents the first example of an all gold‐0 cluster compound besides the only other known gold‐0 compound PPh_3_Au_2_PPh_3_, generated by the reduction of (PPh_3_)AuI with NaC_10_H_8_.^[24]^ These are first intriguing hints towards the existence of gold compounds with all gold atoms in their native oxidation state, especially as besides said examples no further complexes or cluster compounds solely built from gold atoms in the oxidation state of zero are known to date. This aspect is remarkable for the noble metal gold as even within bare transition metals like iron or iridium compounds with an oxidation state of zero of the metal atoms like Fe_3_(CO)_12_ or Ir_4_(CO)_12_ are known.^[25]^

In the following we report the synthesis and structural characterization of an all‐gold‐0 superatomic cluster compound Au_20_(^*t*^Bu_3_P)_8_ solely stabilized by phosphine ligands and exhibiting a high symmetry structure, closely related to bulk gold fcc‐structure. Quantum chemical calculations along with transient absorption measurements give deeper insights into the cluster and corroborate the unusual oxidation state of the gold atoms within this cluster.

The title compound is generated by reduction of ^t^Bu_3_PAuCl with NaBH_4_ in ethanol. The synthetic protocol resembles the previously published Au_32_‐syntheses,^[26]^ solely differing in the employed phosphine ligands and the work‐up procedure used to yield the cluster in crystalline form. After the reaction all volatiles are removed by vacuo to yield a viscous brown residue. Extraction of the crude product with pentane leads to the formation of single crystals of Au_20_(^t^Bu_3_P)_8_ **1** suitable for single crystal X‐ray analysis. The derived structure is displayed in figure 1 (further experimental details and crystallographic data can be found in the Supporting Information).

**Figure 1:**
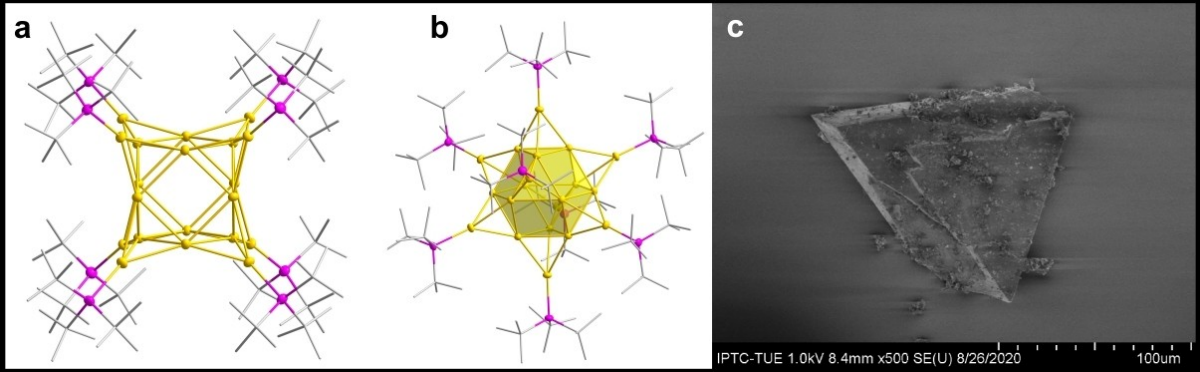
a) Molecular structure of Au_20_(^t^Bu_3_P)_8_ **1**. The ^t^Bu groups are shown by a stick presentation. Thermal ellipsoids set to 50 % (gold atom; yellow, phosphorus atoms; pink). b) Molecular structure of Au_20_(^t^Bu_3_P)_8_ **1** with the central Au_12_ cubocatahedron highlighted by a polyhedral presentation. c) Electron microscopic picture of a single crystal of Au_20_(^t^Bu_3_P)_8_ **1**.

The title compound Au_20_(^t^Bu_3_P)_8_ **1** crystallizes in the space group *R* $\bar{3}$ , with *z*=3. Within the asymmetric unit 4 gold atoms are present. The cluster possesses *O*_h_‐symmetry, showing the high symmetry of the compound. It consists of a central hollow cuboctahedron motif formed by 12 gold atoms surrounded by 8 gold atoms, each bound to a ^*t*^Bu_3_P‐group capping each of the eight triangular faces of the cuboctahedron. The three gold atoms of the triangular faces along with the capping gold atom forming tetrahedrons with all angles being at 60° while angles of the hexagons within the cuboctahedron are 120°.

The structure of the core resembles the face centered cubic (fcc) packing of gold atoms in elemental bulk, solely deviating in the missing central gold atom. The high symmetric and fcc‐like structure of this compound distinguishes this cluster from the sole other example of an all gold‐0 metalloid cluster Au_22_ ^[23]^ which is composed of two phosphine‐bridged Au_11_‐centaur polyhedra, thus not showing bulk gold features. The gold core within **1** has a cubic structure with an edge length of 0.51 nm. The whole compound, including the phosphine ligands, can be approximated as a sphere with a diameter of about 1.9 nm, measured from the outermost hydrogen atoms.

Furthermore, the structure of Au_20_ deviates from the tetrahedral arrangement of the gold atoms predicted by theoretical calculations and proven for gas phase clusters. The stabilizing phosphines may be held accountable for this alternation, as predicted by Häkkinen in 2016.^[27]^

Due to the high symmetry of the cluster all Au−Au bonds are of equal length of 272 pm which is contracted compared to the Au−Au distances of 288 pm found in bulk gold. Comparable gold bond lengths are more likely to be found in smaller gold clusters^[28]^ than in the larger representatives like Au_279_ ^[14]^ where Au−Au‐distances tend to approach bulk lengths. Consequently, the bond length can be described as molecular‐like, which is in contradiction to the metal‐like structure and homogeneity of the bond lengths within the cluster and might be attributed to the missing central gold atom which leads to a shrinkage of the cluster core. Additionally, the missing central gold atom suggests that the stabilization of the cluster can be traced to the bulky phosphine ligands due to sterics. As phosphine ligands are neutral donor molecules, the oxidation state of the Au_20_ core in **1** is zero, being thus one of only two known metalloid gold cluster compounds exhibiting the same oxidation state as the element.

To rule out potential adsorbates, such as protons, and therefore a charge of the cluster molecule, HR‐ESI‐MS measurements were conducted (see Supporting Information, Figure S2). The measurement shows the cluster as double charged cation without any additional adsorbates which substantiates the oxidation state of the gold atoms of ±0. To further support this, XPS measurements were performed (see Supporting Information Figure S4). The measurements yield a signal for the gold 4f_7/2_ at 84.14 eV. This signal is similar to literature values of elemental gold, which range from 84 eV to 84.07 eV.^[29]^ Furthermore, they are clearly distinguished from known signals of 4f_7/2_ orbitals of small gold clusters Au_9_(PPh_3_)_8_(NO_3_)_3_ (85.2 eV) and Au_11_(PPh_3_)_8_I_3_ (84.7 eV), where the oxidation state of the gold atoms is ≠0.^[30]^

However, there will be a certain electronic effect i.e., charge effect of the phosphine ligands on the gold atoms, which explains the small shift of energy compared to elemental gold.^[31]^ The cluster can be described as elemental gold in its native oxidation state stabilized by neutral ligands; hence, removal of those ligands would yield elemental gold without the need of further reducing agents.

To investigate the electronic effect of the ligand on the individual gold atoms in **1**, we performed ab‐initio simulations on the molecular structure of the title compound at the PBE/TZP level, including relativistic effects using Amsterdam Density Functional (ADF) software.^[32]^ A Hirshfeld analysis was utilized to determine the electronic description of gold atoms in the title compound. Previous studies have indicated that the Hirschfeld analysis, which is based on charge density distribution, produces reliable and realistic charge values for gold nanoparticles.^[33]^ Table S2 provides the Hirshfeld analysis data for the phosphine ligand, the gold atoms bound to the phosphine ligand, and the remaining (core) gold atoms. The core gold atoms have an average charge of −0.075 e, while the gold atoms interacting with the ligand have a slightly negative charge (i.e., −0.015 e). The compensation of charge induces a small electronic effect on the gold atom interacting with ligand directly. However, the inner core gold atoms are slightly negative which may be thought of as creating a gradient change of charge from interior to exterior gold atoms.

We studied the electronic structure of **1** to examine its possible molecular‐like behavior, i.e., superatom character. Previous studies have illustrated gold nanoparticles less than 2 nm behave molecule‐like instead of bulk‐like.^[12]^ The molecule‐like electronic structure characteristic arises from the contributions of valence electrons of the metallic core similar to the spherical jellium model for the free electron gas. Here, the superatom electron configuration should be 1S^2^1P^6^1D^10^2S^2^, a magic number within the spherical jellium model.^[34]^ The highest occupied molecular orbital (HOMO) should have S symmetry and the lowest unoccupied molecular orbital should have F symmetry. However, due to the octahedral arrangement of **1**, we see a different arrangement of molecular orbitals (Figure 2). An analysis of the orbital order indicates that the highest molecular orbital (HOMO) has D symmetry, and that the lowest unoccupied molecular orbital (LUMO) has F symmetry. However, the expected HOMO 2S orbital is shifted higher in energy to the LUMO+3. Though there is a rearrangement of orbital order, the electronic structure maintains a closed shell and indicates molecular‐like character. It should be noted that the origin of the orbital rearrangement is due to the octahedral symmetry of **1**.^[35]^ Previous studies on gold systems such as Au_32_(^*n*^Bu_3_P)_12_Cl_8_, have shown that cluster symmetry plays a key role in the electronic structure of a given system and is instrumental in gaining a complete view of superatom behaviour.[^26^, ^36^]

**Figure 2:**
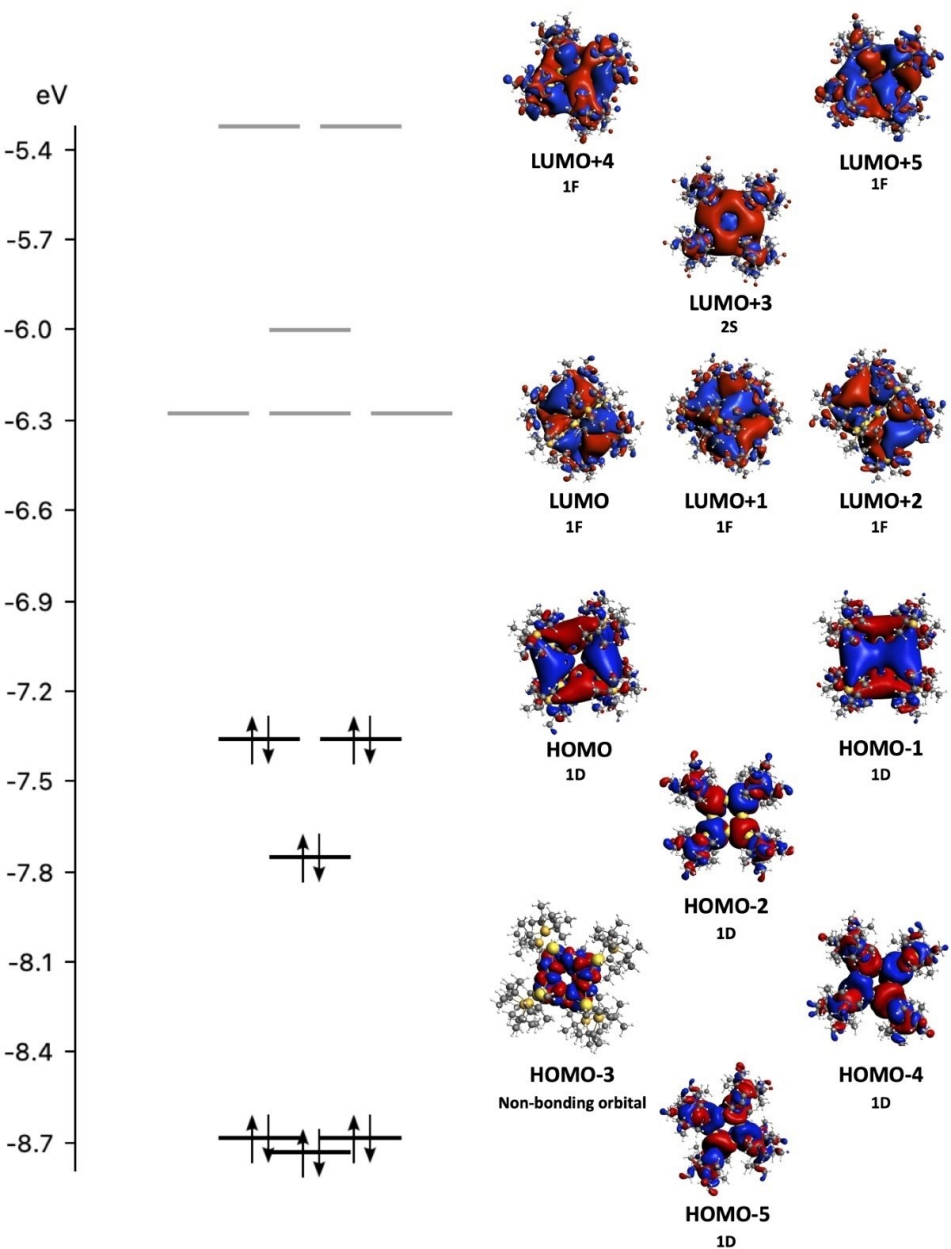
Kohn–Sham orbitals and orbital energy diagram for **1** at the LB94/TZP level of theory.

Absorption spectroscopy of compound **1** reveals several peaks in the UV and Vis spectral range (Figure 3a). The optical transitions are comparable to Au_32_ compounds^[26]^ with a small blue‐shift of all peaks. Differences in optical density could be due to lower extinction coefficients or less solubility, which are not subject of this study.

**Figure 3:**
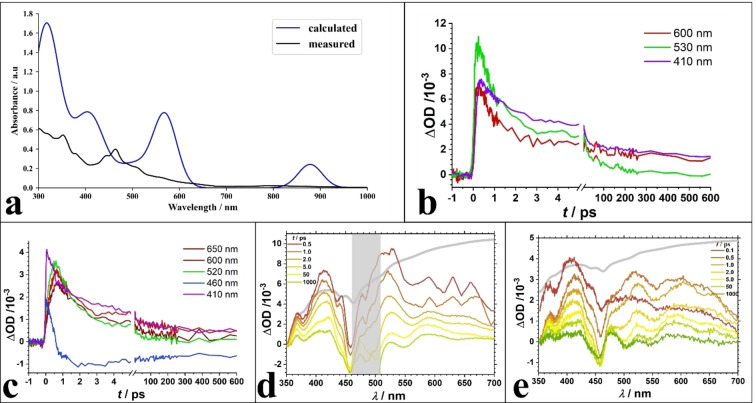
a) Absorption spectra of **1** retrieved both experimentally (black) and theoretically (blue). b, c) exemplary transients at single probe wavelengths of **1** after excitation at 490 nm (b) and 317 nm (c). d, e) Profiles of broadband absorption spectra of compound **1** in di‐fluorobenzene solution after photoexcitation at 490 nm (d) and 317 nm (e) at delay times as indicated. Positive OD values are primarily interpreted as excited state dynamics while negative ones represent ground state bleach. The grey spectrum in (d, e) represents the inverted absorption spectrum while the grey‐shaded vertical bar in (d) indicates scattering due to the excitation pulse.

Time resolved broadband absorption spectra in the UV/Vis wavelength regime further support molecular‐like behavior of compound **1**. For further details of the experimental setup see Supporting Information and elsewhere.^[37]^

The pump wavelength at 490 nm matches the first strong absorption band while 317 nm corresponds to excitation into higher electronic states. As shown in the transient spectra (Figure 3b–e) the low‐energy tail towards the Vis spectral region is negligible owing to stronger extinction coefficients of the higher excited states.

To gain better insight on the origins of the electronic transitions contributing to the prominent peaks, we compared the experimentally obtained UV/Vis spectrum to the simulated one at the PBE/TZP level (details in Supporting Information). The simulated spectrum is red‐shifted from the experimental spectrum, which has been observed in other comparison of gold molecule‐like clusters.^[38]^ We found that the peak around 419 nm is composed mainly of interband transitions originating from atomic states and transitioning to superatomic states (or molecule‐like states). In contrast, the transitions involved at the peak around 320 nm are mainly intraband transitions meaning they originate from and transition to superatomic states. While these transitions are the largest contributors to the peaks at 419 nm and near 320 nm, there are other states that may contribute to the observed transitions. The simulated spectra also point to two other peaks at 571 nm and 877 nm. It is interesting to point out that there is a peak at 877 nm from theoretical simulations. However, this transition is an intraband D to D transition which is forbidden according to the dipole selection rule within the superatom model and thus not observed due to low oscillation strength.

Figure 3d shows transient absorption spectra after 490 nm excitation. Besides ground state bleach around the first absorption band, instantaneous pump‐induced absorption dominates throughout the entire spectral region from 350 to 720 nm. Exemplarily shown single transients reveal multi‐exponential decay (cf. **3 b** and **3 c**), typical for inorganic (metalloid) cluster compounds with prevailing molecular character,^[39]^ that can sufficiently be interpreted as charge localization (sub‐ps time scale), charge recombination (few ps) and recovery of longer‐lived components (hundreds of ps). Inspecting transients in the vicinity of the ground state bleach reveal that 80–90% of the initially excited population decays within several hundreds of picoseconds.

Interestingly, this situation does not change considerably when increasing the pump power by more than 1.3 eV to an excitation wavelength of 317 nm (cf. **3 c** and **3 e**), i.e., when going from inter‐ to intraband excitation according to theory (see above). This implies rather efficient internal conversion processes within the time resolution of the experiment, i.e., within 80 fs or faster.

In summary, we reported an all phosphine stabilized metalloid Au_20_ cluster built solely from gold atoms in the oxidation state 0. Although the molecular characteristics of **1** are expected due to the size, they contrast with the fcc‐like arrangement and oxidation state of the gold atoms. The metallic core structure of **1** is in accordance with theoretical predictions, along with its high symmetry. The deviation from the tetrahedral arrangement is presumably caused by the phosphine ligands.

As previous studies have shown, the stabilizing phosphine ligands have great impact upon the resulting cluster structures.[^26^, ^27^, ^40^] Why or how the chosen ^*t*^Bu_3_P enables the presented structure and the unusual oxidation state of the gold atoms remains elusive and needs to be further studied. Especially further investigations must show if other metalloid cluster compounds of gold in oxidation state of zero are accessible, opening a new area of metalloid gold clusters which is directly correlated to the elemental state without a redox chemistry. This might lead to the possibility to dissolve elemental gold and obtain elemental gold from molecular precursors without a redox reagent.

## Author Contributions

F.F. developed and executed the cluster synthesis. N.P. and A.C. performed and evaluated the quantum chemical calculations. N.C.M. and A.N.U. performed and evaluated the transient absorption measurements. MS conducted and interpreted the XPS‐measurements. F.F. and N.P. interpreted the results and wrote the manuscript. A.S. conceived and supervised the project. All authors have given approval to the final version of the manuscript.

## Conflict of interest

The authors declare no conflict of interest.

## Supporting information

As a service to our authors and readers, this journal provides supporting information supplied by the authors. Such materials are peer reviewed and may be re‐organized for online delivery, but are not copy‐edited or typeset. Technical support issues arising from supporting information (other than missing files) should be addressed to the authors.

Supporting Information

Supporting Information

Supporting Information

## Data Availability

The data that support the findings of this study are available in the supplementary material of this article.
