# Supplementary material for: Au20( t Bu3P)8: A Highly Symmetric Metalloid Gold Cluster in Oxidation State 0
Source: Angew Chem Int Ed Engl. 2022 Jul 28;61(36):e202206019. doi: 10.1002/anie.202206019 (PMC9546110; doi:10.1002/anie.202206019)

checkCIF/PLATON report

Structure factors have been supplied for datablock(s) 57_sq

THIS REPORT IS FOR GUIDANCE ONLY. IF USED AS PART OF A REVIEW PROCEDURE FOR PUBLICATION, IT SHOULD NOT REPLACE THE EXPERTISE OF AN EXPERIENCED CRYSTALLOGRAPHIC REFEREE.

No syntax errors found. CIF dictionary Interpreting this report

Datablock: 57_sq

Bond precision: C-C = 0.0177 A Wavelength=0.71073

Cell: a=19.2061(9) b=19.2061(9) c=33.727(2) alpha=90 beta=90 gamma=120

Temperature: 150 K

	Calculated	Reported
Volume	10774.2(12)	10774.1(12)
Space group	R -3	R -3
Hall group	-R 3	-R 3
Moiety formula	C96 H216 Au20 P8 [+ solvent]	C96 H216 Au20 P8
Sum formula	C96 H216 Au20 P8 [+ solvent]	C96 H216 Au20 P8
Mr	5557.84	5557.77
Dx,g cm-3	2.570	2.570
Z	3	3
Mu (mm-1)	20.452	20.452
F000	7476.0	7476.0
F000’	7374.90	
h,k,lmax	23,23,41	23,23,41
Nref	4726	4723
Tmin,Tmax	0.328,0.382	0.581,0.746
Tmin’	0.162	

Correction method= # Reported T Limits: Tmin=0.581 Tmax=0.746
AbsCorr = MULTI-SCAN

Data completeness= 0.999 Theta(max)= 25.998

R(reflections)= 0.0231(4031) wR2(reflections)= 0.0541(4723)

S = 1.091 Npar= 296

The following ALERTS were generated. Each ALERT has the format
test-name_ALERT_alert-type_alert-level. Click on the hyperlinks for more details of the test.

Alert level B

```
PLAT094_ALERT_2_B Ratio of Maximum / Minimum Residual Density ....       4.65 Report
```

… most likely due to a non-perfect absorption coorection, especially since the highest electron density is located at the special position 0 0 0 (at the center of the molecule) and no chemically meaningful atom could be refined there.

Alert level C

```
PLAT242_ALERT_2_C Low    'MainMol' Ueq as Compared to Neighbors of         P3 Check
PLAT242_ALERT_2_C Low    'MainMol' Ueq as Compared to Neighbors of         P4 Check
PLAT342_ALERT_3_C Low Bond Precision on  C-C Bonds ...............    0.01767 Ang.
PLAT412_ALERT_2_C Short Intra XH3 .. XHn      H30A      ..H30F      .       1.87 Ang.
                                                -x+y,1-x,z  =      3_565 Check
PLAT923_ALERT_1_C S    Values in the CIF and FCF Differ by .......      0.016 Check
PLAT973_ALERT_2_C Check Calcd Positive Resid. Density on       Au2        1.12 eA-3
```

Alert level G

```
PLAT003_ALERT_2_G Number of Uiso or Uij Restrained non-H Atoms ...         28 Report
PLAT083_ALERT_2_G SHELXL Second Parameter in WGHT  Unusually Large     186.43 Why ?
PLAT178_ALERT_4_G The CIF-Embedded .res File Contains SIMU Records          2 Report
PLAT186_ALERT_4_G The CIF-Embedded .res File Contains ISOR Records          3 Report
PLAT187_ALERT_4_G The CIF-Embedded .res File Contains RIGU Records          2 Report
PLAT301_ALERT_3_G Main Residue  Disorder ..............(Resd  1  )        58% Note
PLAT605_ALERT_4_G Largest Solvent Accessible VOID in the Structure        167 A**3
PLAT811_ALERT_5_G No ADDSYM Analysis: Too Many Excluded Atoms ....          ! Info
PLAT860_ALERT_3_G Number of Least-Squares Restraints .............        282 Note
PLAT869_ALERT_4_G ALERTS Related to the Use of SQUEEZE Suppressed           ! Info
PLAT910_ALERT_3_G Missing # of FCF Reflection(s) Below Theta(Min).          3 Note
PLAT913_ALERT_3_G Missing # of Very Strong Reflections in FCF ....          1 Note
PLAT933_ALERT_2_G Number of OMIT Records in Embedded .res File ...          3 Note
PLAT960_ALERT_3_G Number of Intensities with I  <   - 2*sig(I) ...          1 Check
PLAT978_ALERT_2_G Number C-C Bonds with Positive Residual Density.          1 Info
```

---

0 **ALERT level A** = Most likely a serious problem - resolve or explain
1 **ALERT level B** = A potentially serious problem, consider carefully
6 **ALERT level C** = Check. Ensure it is not caused by an omission or oversight
15 **ALERT level G** = General information/check it is not something unexpected

1 ALERT type 1 CIF construction/syntax error, inconsistent or missing data
9 ALERT type 2 Indicator that the structure model may be wrong or deficient
6 ALERT type 3 Indicator that the structure quality may be low
5 ALERT type 4 Improvement, methodology, query or suggestion
1 ALERT type 5 Informative message, check

It is advisable to attempt to resolve as many as possible of the alerts in all categories. Often the minor alerts point to easily fixed oversights, errors and omissions in your CIF or refinement strategy, so attention to these fine details can be worthwhile. In order to resolve some of the more

serious problems it may be necessary to carry out additional measurements or structure refinements. However, the purpose of your study may justify the reported deviations and the more serious of these should normally be commented upon in the discussion or experimental section of a paper or in the "special_details" fields of the CIF. checkCIF was carefully designed to identify outliers and unusual parameters, but every test has its limitations and alerts that are not important in a particular case may appear. Conversely, the absence of alerts does not guarantee there are no aspects of the results needing attention. It is up to the individual to critically assess their own results and, if necessary, seek expert advice.

Publication of your CIF in IUCr journals

A basic structural check has been run on your CIF. These basic checks will be run on all CIFs submitted for publication in IUCr journals (*Acta Crystallographica*, *Journal of Applied Crystallography*, *Journal of Synchrotron Radiation*); however, if you intend to submit to *Acta Crystallographica Section C* or *E* or *IUCrData*, you should make sure that full publication checks are run on the final version of your CIF prior to submission.

Publication of your CIF in other journals

Please refer to the *Notes for Authors* of the relevant journal for any special instructions relating to CIF submission.

PLATON version of 16/07/2020; check.def file version of 12/07/2020

Datablock 57_sq - ellipsoid plot

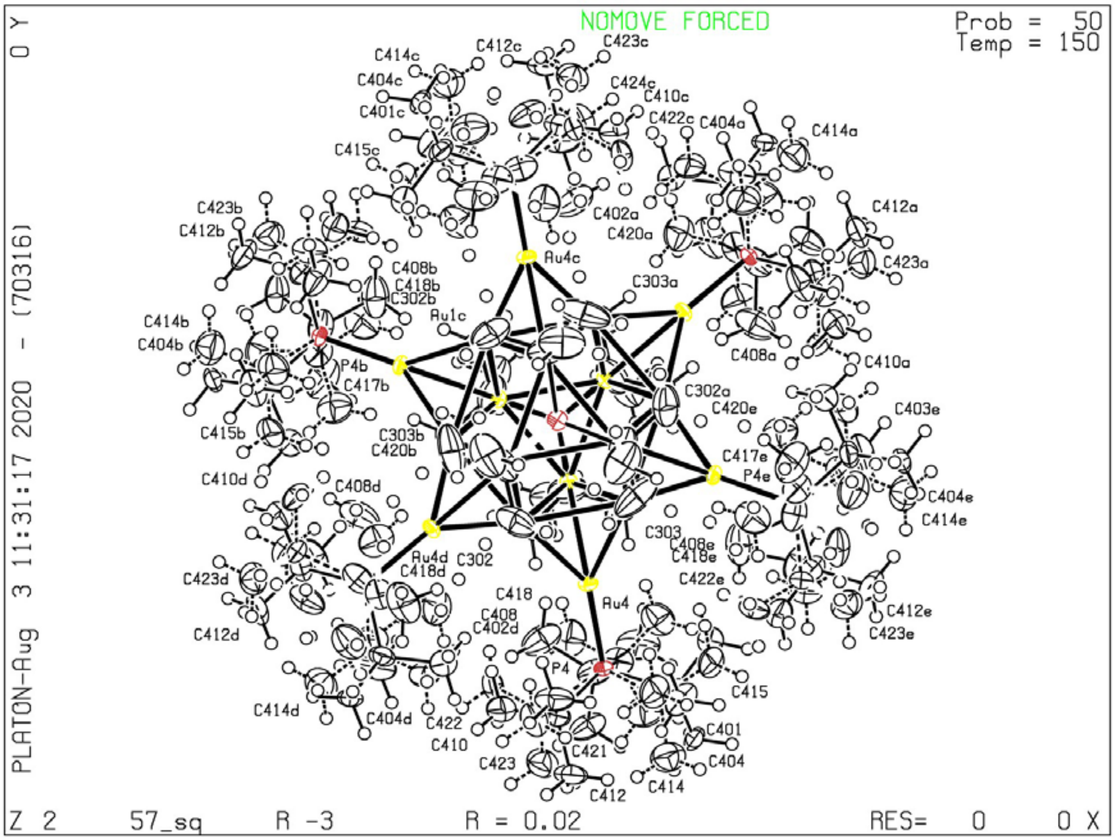

Supplement: Supplementary file 2 — Supporting Information [file ANIE-61-0-s003.pdf]
