# Supplementary material for: Au20( t Bu3P)8: A Highly Symmetric Metalloid Gold Cluster in Oxidation State 0
Source: Angew Chem Int Ed Engl. 2022 Jul 28;61(36):e202206019. doi: 10.1002/anie.202206019 (PMC9546110; doi:10.1002/anie.202206019)
Supplement: Supplementary file 3 — Supporting Information [file ANIE-61-0-s001.pdf]

Angewandte Chemie
Eine Zeitschrift der Gesellschaft Deutscher Chemiker

Supporting Information

$Au_{20}(^{t}Bu_3P)_8$: A Highly Symmetric Metalloid Gold Cluster in Oxidation State 0

F. Fetzer, N. Pollard, N. C. Michenfelder, M. Strienz, A. N. Unterreiner, A. Z. Clayborne, A. Schnepf**

Supporting Information

1. Synthesis of $Au_{20}(^tBu_3P)_8$

434 mg of $^tBu_3PAuCl$ (1 mmol) is dissolved in 15 ml of ethanol before 38 mg of $NaBH_4$ (1 mmol) in 20 ml of ethanol is added. The reaction is stirred for one hour before all volatiles are removed in vacuo. The crude product is dissolved in pentane and stored at room temperature. Black crystals of $Au_{20}(^tBu_3P)_8$ **1** are formed overnight.

Yield in respect to gold: 9 mg, 1.5 µmol, 0.15%

2. SEM Images of crystals of $Au_{20}(^tBu_3P)_8$

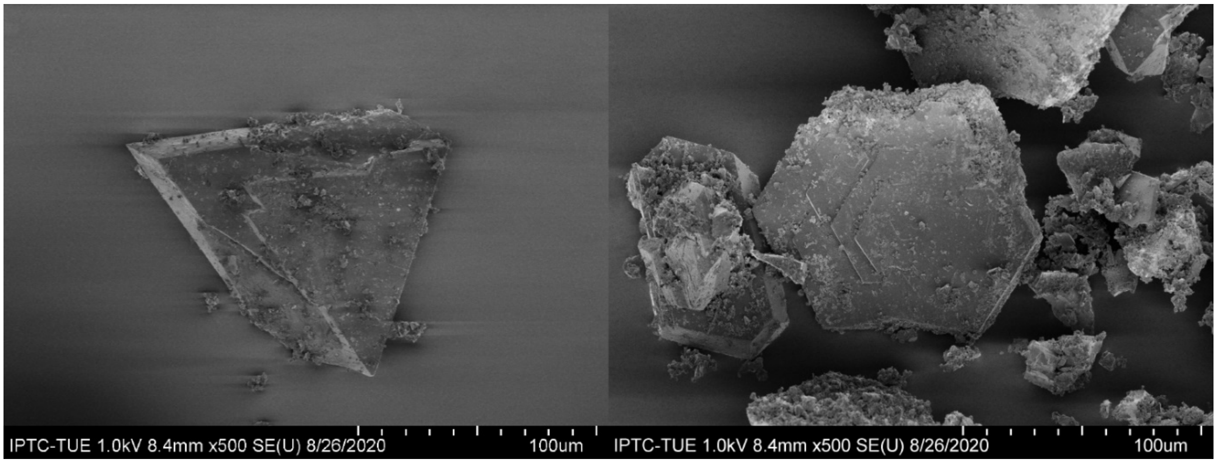

Figure S1: High-resolution SEM micrograph of Au_{20}-NC micro-crystals.

3. Elemental Analysis

Table S1: Elemental Analysis of $Au_{20}(^tBu_3P)_8$. Single crystal X-ray analysis shows three clusters and 18 molecules of ethanol per unit cell. Accordingly, the EA was calculated for $[Au_{20}C_{106}H_{216}P_8][C_2H_5OH]_6$.

calculated	C: 22.23 %	H: 4.35 %
measured	C: 22.03 %	H: 4.22 %

4. HR-ESI-MS

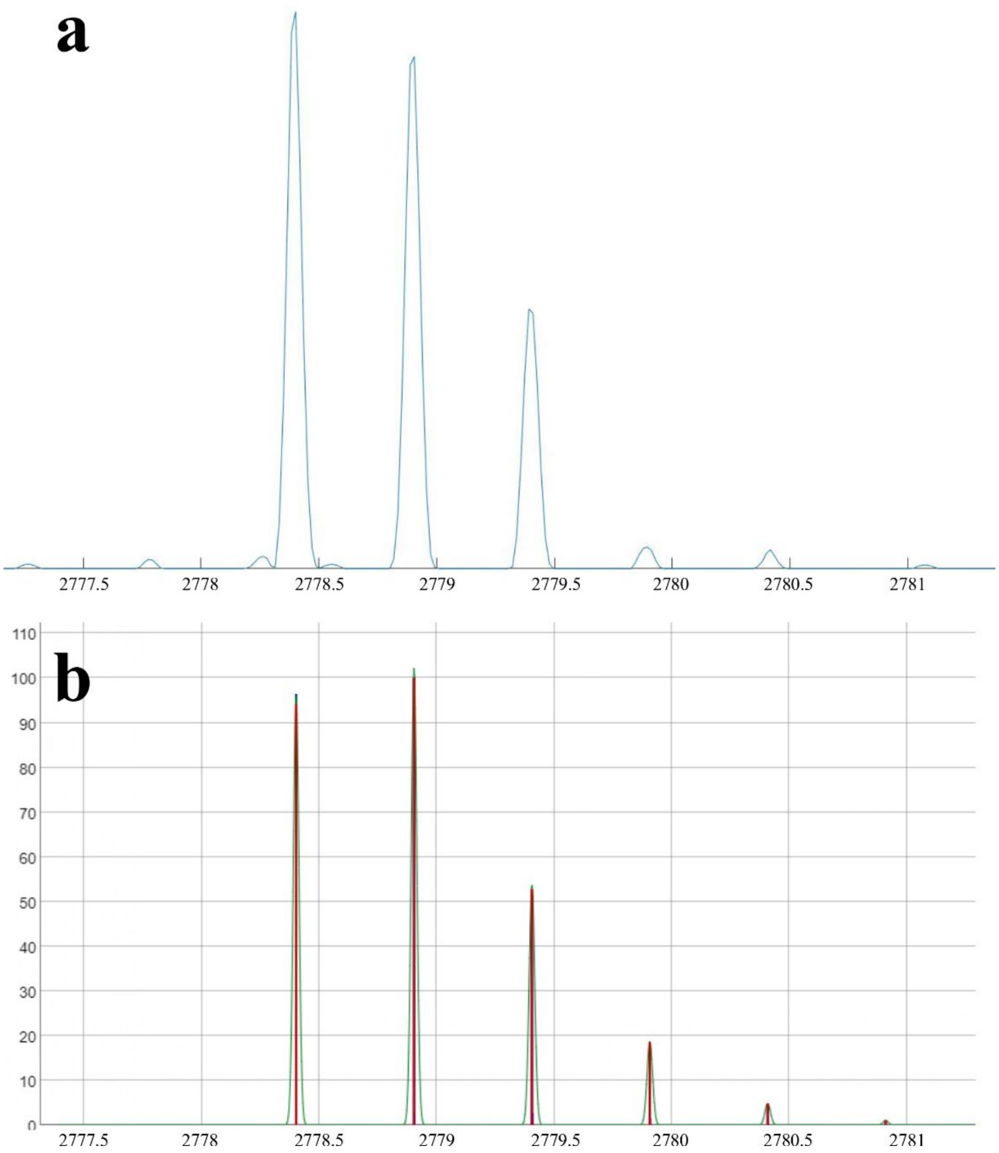

Figure S2: HR-ESI-MS spectrum of dissolved crystals of $Au_{20}(^tBu_3P)_8$ in dichloromethane. **a)** Measured spectrum. **b)** simulated spectrum. Compound **1** is observed as a doubly charged cation at m/z = 2778.95 with z = 2 corresponding to the molar mass of **1** of M = 5557.8911 g/mol.

5. Computational Details

First-principle density functional calculations were implemented using a local version of the Amsterdam Density Functional (ADF) 2020.03 program.[1] Structural relaxation was performed

using the structure determined by X-ray analysis of **1**. The exchange and correlation effects were accounted for through the gradient corrected Perdew, Burke, and Ernzerhof (PBE)[2] functional, which has been utilized in previous studies to give accurate results for small to medium-sized gold clusters. [3] A triple-z with polarization functions (TZP) Slater type basis set [4] was utilized and the zeroth-order regular approximation (ZORA) was employed in the calculations to account for the scalar relativistic effects. [5] Bond lengths were calculated using ADF and are shown below in Table S1. We found that the bond lengths of **1** differ by only 2.57% as compared to the crystal structure obtained from experiment. Visualization of the electron density was also rendered using ADF.

Table S2: Comparison of bond lengths (Å) between the relaxed structure (**1**) and the unrelaxed crystal structure of $Au_{20}(^tBu_3P)_8$

**Bonds**	**$Au_{20}(^tBu_3P)_8$**	**Experimental**
Au-Au	2.79 Å	2.72 Å
Au-P	2.35 Å	2.30 Å

The simulated optical spectra were obtained using linear response-time dependent density functional theory (Lr-TDDFT)[6] as implemented in ADF using the PBE functional. We obtained the optical spectra for a reduced version of **1** ($Au_{20}(CH_3P)_8$) without solvent effects.

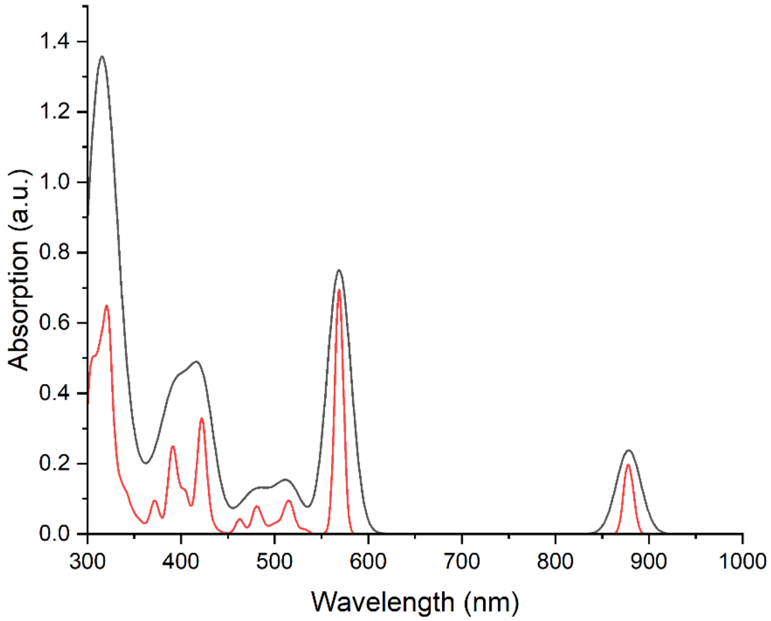

Figure S3: Theoretical Absorption Spectra of **1** using both a broad (30) and fine (10) width.

Table S3: Hirschfeld Charge Analysis of **1.**

Core Au (e)	Ligand Au (e)	Ligand (PR_3) (e)
-0.074	0.016	0.095
-0.077	0.013	0.097
-0.076	0.016	0.097
-0.075	0.014	0.098
-0.076	0.014	0.094
-0.074	0.016	0.10
-0.074	0.013	0.099
-0.076	0.016	0.099
-0.075		
-0.076		
-0.076		
-0.074		

6. Transient absorption spectra

A very small portion of the 800 nm (Astrella (Coherent), 7 mJ, 35 fs, repetition rate 1 kHz) laser output was focused into a movable 2 mm CaF2 crystal (nortus optronic GmbH) to generate a white-light continuum between 350 and 720 nm. After passing the sample and a fused silica prism, the white-light was spectrally expanded and recorded by a CCD Camera (Series 2000, Si Photodetector, Entwicklungsbüro Stresing). A 400 nm pumped noncollinearly optical-parametric amplifier (NOPA) generated pump wavelengths at 490 and – after frequency doubling of 640 nm NOPA pulses – the second pump wavelength of 317 nm. The spot size in the sample was about 200 µm, which was more than twice the white-light spot size. Excitation energies were typically 400 nJ per pulse. The delay of the pump pulses was accomplished by a computer-controlled translation stage (maximum delay ~1.2 ns, Thorlabs), whereby every second pulse was blocked with an optical chopper (Thorlabs) resulting in transient spectra with and without excitation. Differentiation results in ΔOD spectra with a time resolution better than 100 fs. Data were collected and stored using an in-house written Labview program.

7. Thin film preparation

The thin films were prepared by dropcasting of 50 µl of a 0.45 µM solution of **1** in THF onto a 0.75x0.75 cm silicon wafer in a glovebox. The silicon wafer has been pre-cleaned with Propan-2ol and acetone in an ultrasonic bath for 3 minutes respectively. After drying for 1 minute, the sample was spin coated for 1 minute at 10 rps.

8. XPS Measurements

XPS measurements were carried out in a multichamber UHV system (base pressure 8x10^-10 mbar) using a Phoibos 100 MCD electron analyzer and a 1d-Delay Line detector (SPECS). Mg-K_α radiation from a Al/Mg Anode (XR-50m x-ray source, 1253.64 eV) was used. The survey spectra were collected using the following parameters: 50 eV pass energy, 0.2 s dwell time and 0.5 eV step width for 3 accumulations. The spectra of the Au4f region and the P2p region were collected with the following parameters: 20 eV pass energy, 0.5 s dwell time and 0.05 eV step width for 5 accumulations. The spectral analysis was done with the software Unifit version 2018 (Unifit Scientific Software GmbH).[7]

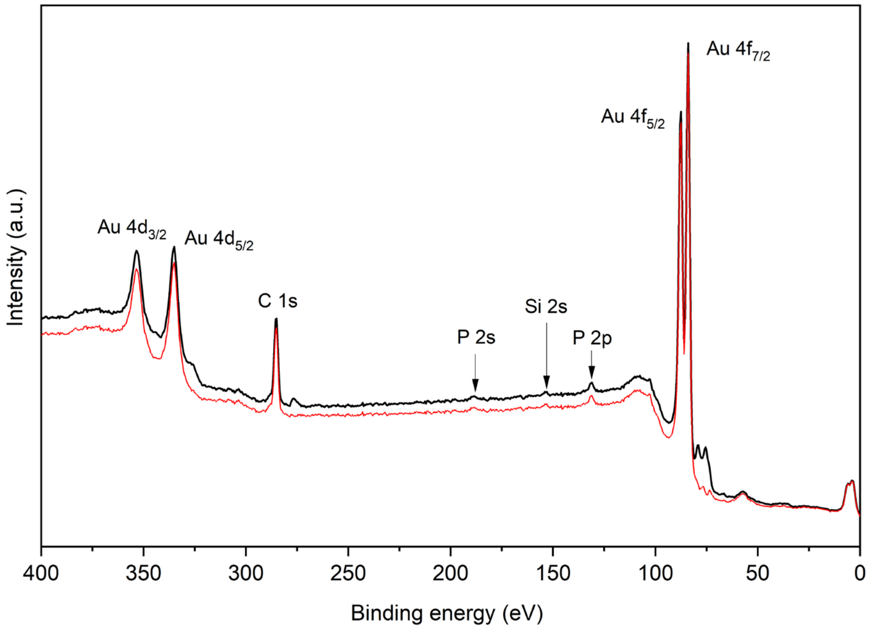

Figure S4: Survey XPS-Spectra of **1** from 0 to 400 eV (black) and without satellite peaks (red). The signal of the Au $4f_{7/2}$ orbitals can be found at 84.14 eV.

9. Crystallographic Data

Formula	$C_{96}H_{216}Au_{20}P_8$
Formula weight	5557.77
T /K	150.01
Crystal system	trigonal
Space group	R-3
a /Å	19.2061(9)
b /Å	19.2061(9)
c /Å	33.727(2)
α /°	90
β /°	90
γ /°	120
V /Å^3	10774.1(12)
Z	3
μ /mm^{-1}	20.452
ρ /g·cm^{-3}	2.570
Reflections collected	96935
Independent reflections	4723
R(int.)	0.0696
GooF	1.091
R_1 (I > 2σ)	0.0231
wR_2 (all data)	0.0541
CCDC number	2162483

[1] G. te Velde, F. M. Bickelhaupt,E. J. Baerends, C. Fonseca Guerra, S. J. A. van Gisbergen, J. G. Snijders, T. Ziegler, *J. Comput. Chem.* **2001**, *22*, 931.
[2] John P. Perdew, Kieron Burke, Matthias Ernzerhof, *Phys. Rev. Lett.* **1996**, *77*.
[3] S. Kenzler, F. Fetzer, C. Schrenk, N. Pollard, A. R. Frojd, A. Z. Clayborne, A. Schnepf, *Angew. Chem. Int. Ed.* **2019**, *58*, 5902.
[4] E. van Lenthe, E. J. Baerends, *Journal of computational chemistry* **2003**, *24*, 1142.
[5] a) E. van Lenthe, R. van Leeuwen, E. J. Baerends, J. G. Snijders, *Int. J. Quantum Chem.* **1996**, *57*, 281; b) E. van Lenthe, E. J. Baerends, J. G. Snijders, *J. Chem. Phys.* **1994**, *101*, 9783; c) E. van Lenthe, A. Ehlers, E.-J. Baerends, *J. Chem. Phys.* **1999**, *110*, 8943.
[6] J. Yan, J. J. Mortensen, K. W. Jacobsen, K. S. Thygesen, *Phys. Rev. B* **2011**, *83*.
[7] R. Hesse, T. Chassé, P. Streubel, R. Szargan, *Surf. Interface Anal.* **2004**, *36*, 1373.
